# Foot Strike Patterns During a Continuous 1600 m Outdoor Run in Recreational Female Runners

**DOI:** 10.3390/sports14100453

**Published:** 2026-10-13

**Authors:** Daniel Scherrer, Kylie A. Legg, Chris W. Rogers, Darryl J. Cochrane

**Affiliations:** 1 School of Sport, Exercise and Nutrition, Massey University, Palmerston North 4474, New Zealand; d.cochrane@massey.ac.nz; 2 School of Veterinary Science, Massey University, Palmerston North 4474, New Zealand; k.legg@massey.ac.nz (K.A.L.); c.w.rogers@massey.ac.nz (C.W.R.)

**Keywords:** foot strike, running gait, middle-distance running

## Abstract

Foot strike pattern is typically classified from a single observation, which may not capture how it changes during a continuous run. This study examined how the proportion of heel strikes changed across a continuous 1600 m outdoor track run, completed at a self-selected maximal effort by 20 recreational female runners, and its associations with running speed, heart rate, cadence and contact time. Vertical ground reaction force was recorded from the right foot with a force-sensing insole sampling at 200 Hz. Every stride was classified as a heel, midfoot or forefoot strike from the morphology of the vertical force-time curve. Odds of heel relative to midfoot striking were estimated with binary logistic mixed-effects models and expressed as odds ratios (OR). The proportion of heel strikes did not change systematically across the four laps. Longer contact time was associated with lower odds of heel striking (OR 0.988), whereas higher heart rate relative to a runner’s own mean (OR 1.047) and faster running speed (OR 1.277) were associated with greater odds (all *p* < 0.001). Cadence was not associated with foot strike. Differences between runners accounted for most of the explained variance (marginal *R*^2^ = 0.18, conditional *R*^2^ = 0.81). A single observation may therefore not represent the foot strike pattern used across a whole run.

## 1. Introduction

Runners typically contact the ground with the heel, midfoot, or forefoot during the initial phase of stance. Foot strike pattern influences how ground reaction forces are distributed at landing. Heel striking produces a distinct impact transient within the first 50 ms of stance that is largely absent during midfoot and forefoot striking [1]. At matched speeds, heel striking produces higher loading rates and peak impact forces than forefoot striking [2]. Forefoot striking is associated with greater ankle loading, whereas heel striking is associated with greater knee loading [2]. Most recreational runners habitually adopt this higher-loading pattern [3]. However, foot strike is not constant, as runners may alter their strike pattern in response to running speed, surface, and footwear [4].

Runners in long-distance road races are predominantly heel strikers [3]. The prevalence of heel striking increases further as race distance increases. The prevalence of heel striking increased from approximately 89% at 10 km to 93% at 32 km during a road race [5]. In a 15 km road race, the prevalence of heel striking increased by 14% between 3 km and 13 km [6]. Consistent with these findings, a meta-analysis of 12 studies reported that approximately 11% of runners change foot strike pattern during a race, with the majority transitioning from a non-heel to a heel strike as the race progressed [3]. Foot strike pattern therefore remains stable in most runners, whereas the increase in heel strike prevalence reflects a minority who transition from a non-heel to a heel strike. Fewer data are published at middle distances, and what exists comes from single-lap observation. Foot strike was classified once per runner during 800 m and 1500 m races, with 27% heel, 42% midfoot and 31% forefoot striking [7]. Across a maximal 800 m effort on an outdoor track, no change in foot strike proportion was observed in twenty-one runners monitored with a foot-mounted inertial measurement unit [8]. Additionally, at 1500 m, lap-by-lap changes in contact time and cadence have been reported, although foot strike pattern was not classified [9].

Current evidence is also constrained by how foot strike pattern has been measured. Most field-based studies have classified foot strike from video recordings obtained at fixed locations during a race [5], providing only discrete time points rather than continuous assessment throughout a run [10]. Comparing two time points shows whether the prevalence of heel striking differed between them but cannot identify which runners changed or when the change occurred. Laboratory studies have used instrumented treadmills, although treadmill running alters kinematics at foot strike relative to overground running [11]. Wearable sensor technology has the potential to overcome these limitations. However, a scoping review of 231 wearable sensor-based running gait studies found that 72% were conducted indoors and 67% analysed less than 200 m of running [12]. In-shoe force sensors can classify foot strike pattern during overground running [13], and per-step classification has been achieved outdoors using accelerometry [14]. Foot strike has not, however, been classified from in-shoe force on every stride of a continuous middle-distance run analysed by lap. The populations studied also matter, with heel striking associated with higher vertical loading rates in female than male runners [15]. Female runners also remain under-represented in running biomechanics research, with only 6% of sport and exercise science publications recruiting female-only cohorts [16].

Understanding how foot strike changes during continuous overground outdoor running may improve our understanding of gait adaptations during sustained middle-distance running. This study aimed to examine how the proportion of heel strikes changed over four successive 400 m laps of a continuous 1600 m outdoor track run in recreational female runners. Associations between foot strike pattern, running speed, heart rate, cadence and contact time were also examined. It was hypothesised that foot strike pattern would change over the course of the 1600 m run. It was further hypothesised that each of these variables would be independently associated with the adopted foot strike pattern.

## 2. Materials and Methods

### 2.1. Participants

Twenty-one recreational female runners were recruited from the local running community through flyers and word of mouth. Inclusion criteria required participants to train at least three times per week, be able to comfortably run for at least twenty minutes, and be free from injury for at least three months before testing. Written informed consent was obtained from all participants, and ethics approval was granted by the Massey University Human Ethics Committee (OM1 25–42).

A priori power analysis was conducted to estimate the required sample size to detect changes in foot strike pattern and spatiotemporal variables across successive 400 m laps. As no study has examined these variables across a continuous 1600 m run, the effect size was derived from the most comparable study, in which spatiotemporal variables were measured within runners across increasing running distance [17]. A conservative expected effect size was used (Cohen’s *f* = 0.39), with a significance level (α) of 0.05 and statistical power (1 − β) of 0.97. The analysis indicated a minimum sample size of 18 participants (G*Power, version 3.1.9.7, Heinrich-Heine University, Düsseldorf, Germany).

### 2.2. Instrumentation and Testing Procedures

Data collection took place on an outdoor 400 m track (Mondo Super X Performance surface, Mondo Worldwide, Gallo d’Alba, Italy). Environmental conditions during the testing period were ambient air temperature 14.8 ± 2.7 °C, relative humidity 75.1 ± 9.3%, and wind speed 29.2 ± 8.8 km·h^−1^.

Testing involved a single data collection session. Each participant first completed a five-minute standardised warm-up consisting of running at 10 km·h^−1^ on the track. Immediately before testing, a validated force-sensing insole (Loadsol^®^ Pro, Novel GmbH, Munich, Germany) sampling at 200 Hz was inserted into each participant’s shoes [18]. Ground reaction force data were recorded from the right foot throughout the 1600 m run. Running speed and heart rate were recorded at 1 Hz using a wrist-mounted multisport watch with integrated global positioning system capability and a paired chest-mounted heart rate sensor (Polar V800 and H10, Polar Electro Oy, Kempele, Finland). Participants were instructed to complete a continuous 1600 m run at a self-selected maximal effort pace. Participants ran in their own habitual running shoes. Participants answered the question ‘What is your typical foot striking method?’ by selecting heel, midfoot or forefoot, after each pattern had been explained verbally. Self-reported foot strike was compared with the pattern that accounted for most of each runner’s strides.

### 2.3. Signal Processing and Foot Strike Classification

The raw force signal was low-pass filtered using a fourth-order, zero-lag Butterworth filter with a cutoff frequency of 50 Hz. Initial contact was defined as the first point at which the vertical ground reaction force exceeded a threshold of 20 N [19], and toe-off was identified when the force fell below 20 N. The continuous 1600 m running bout was trimmed to steady-state running by evaluating successive ten-stride windows at the start and end of the bout for the coefficient of variation (CV) of stride duration and active peak force. Strides were excluded from each end until the CV fell below 5% for stride duration and 10% for active peak force, with a minimum of five strides removed at each end. Trimming removed 10 to 27 strides per participant, equivalent to 1.2 to 4.8% of the bout.

**Figure 1:**
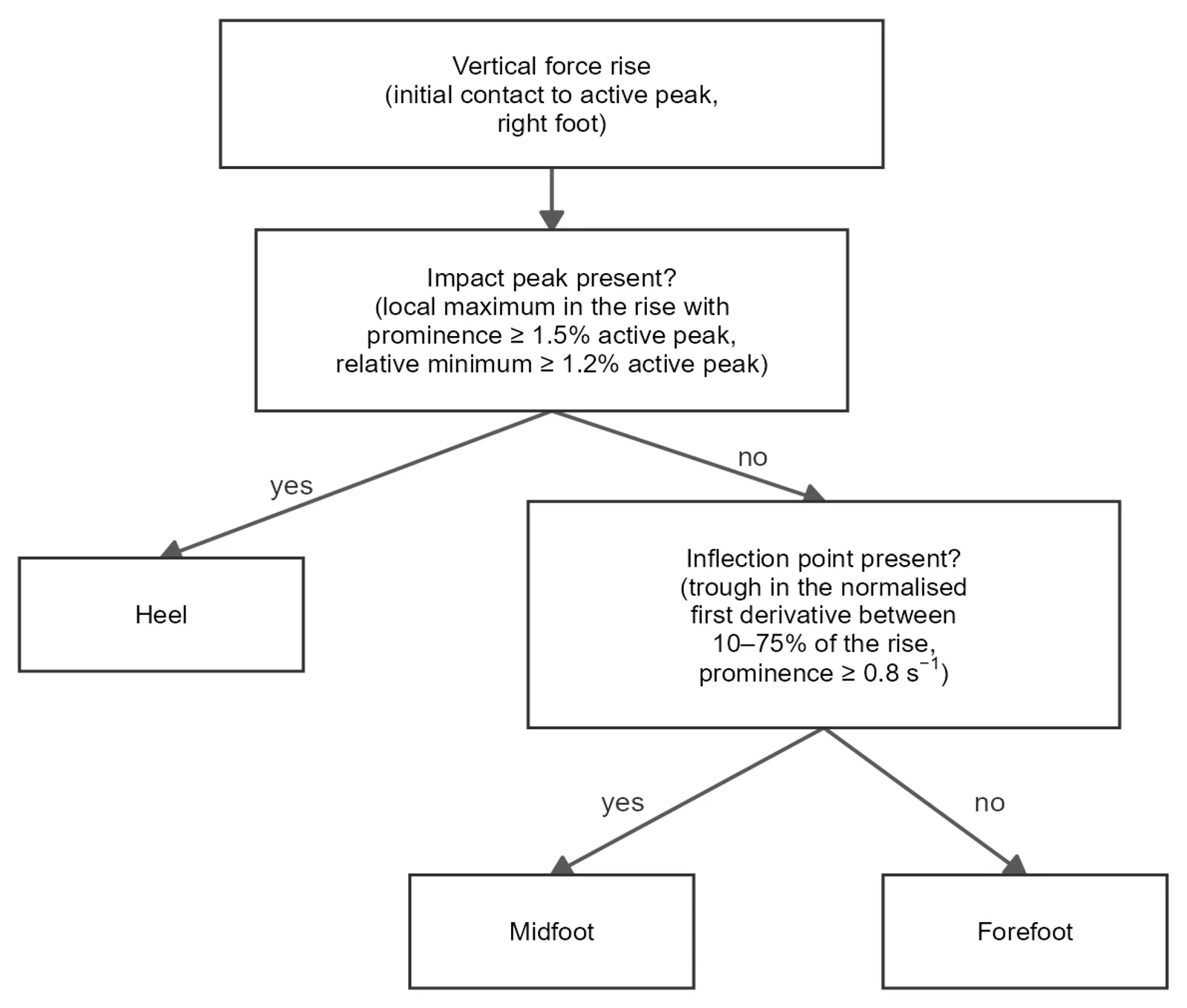
Decision tree for per-stride foot strike classification.

**Figure 2:**
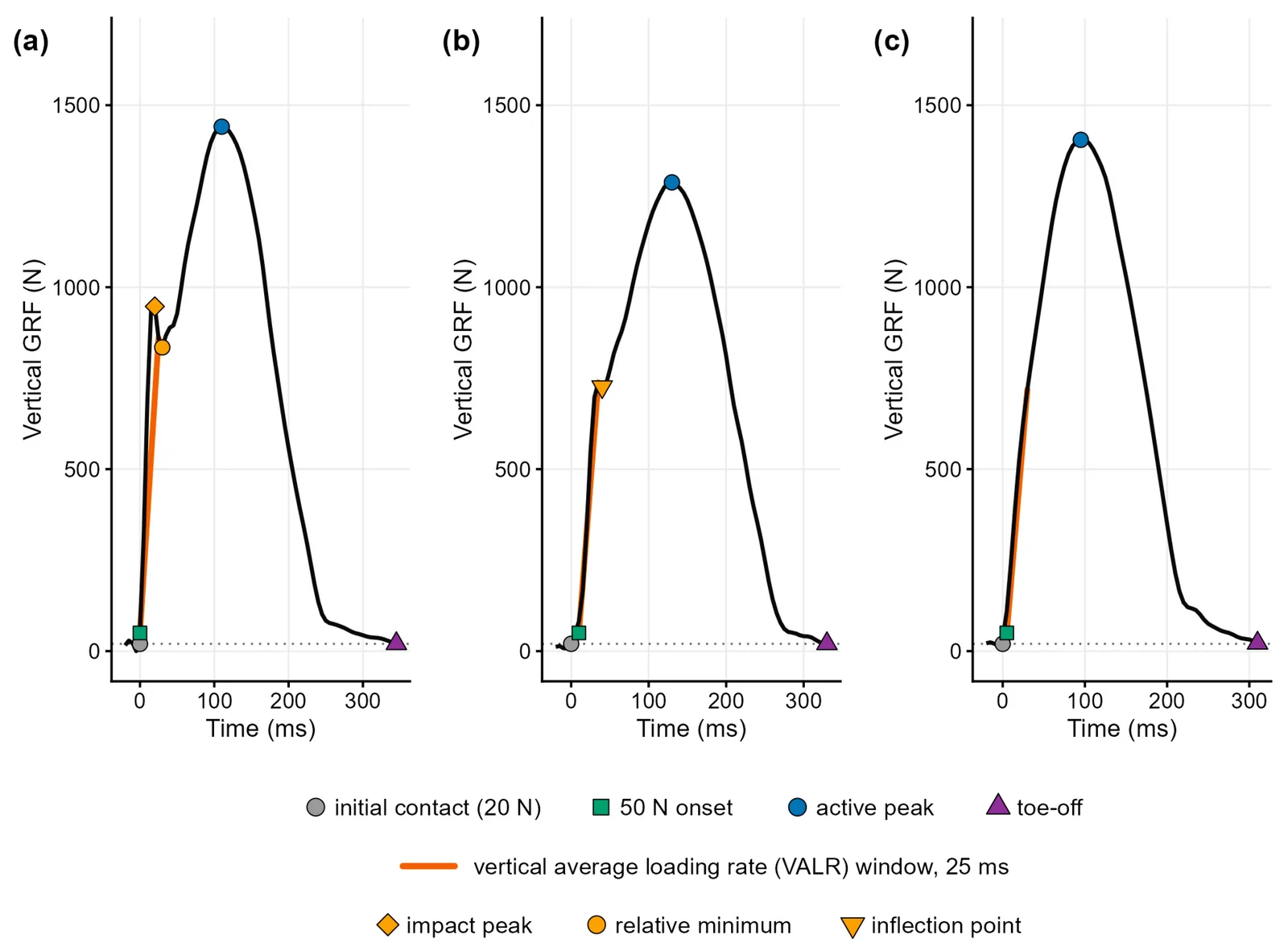
Representative vertical ground reaction force traces. (**a**) Heel: an impact peak followed by a relative minimum. (**b**) Midfoot: an inflection point with no impact peak. (**c**) Forefoot: neither feature. The dotted line marks the 20 N threshold used to define initial contact and toe-off.

For each retained stride, kinetic features, including active peak force, peak impact force, vertical average loading rate (VALR), and vertical impulse, were computed from the force signal as described in a previous study [20]. Foot strike pattern was then classified on a per-stride basis using an algorithmic approach (Figure 1). The algorithm classified foot strike from the shape of the vertical force rise between initial contact and the active force peak, which differs between foot strike patterns [21]. A heel strike produces a distinct impact transient in early stance. This transient is reduced or absent in midfoot and forefoot striking [22]. A true impact peak was defined as an early local maximum exceeding 1.5% of the active peak, followed by a relative minimum at least 1.2% of the active peak below it (Figure 2a). Accordingly, a stride was classified as a heel strike if the force rise exhibited a true impact peak [1], whereas a stride with no impact peak but a detectable inflection point was classified as a midfoot strike. An inflection point was defined as a local trough in the first derivative of the force signal normalised to the active peak, located within 10% to 75% of the initial impact slope, with a prominence of at least 0.8 s^−1^ (Figure 2b). Finally, a stride was classified into a forefoot strike if neither an impact peak nor an inflection point was detected, indicating a smooth, monotonic rise to the active peak (Figure 2c).

### 2.4. Statistical Analysis

Running speed, cadence, and contact time were summarised for each lap and participant, and foot strike pattern was displayed as the ordered sequence of strides within each lap. A principal component analysis was performed on the four continuous running and physiological measures (running speed, heart rate, cadence, and contact time), z-scored across pooled quality strides. The purpose was to describe the correlation structure among these measures and to examine whether they separated heel from midfoot strides. Due to the low frequency of forefoot strikes, a binary logistic mixed-effects model was used to estimate the odds of heel strike (coded 1) relative to midfoot strike (coded 0), with odds ratios calculated as e^β.

Candidate predictors were specified a priori and comprised lap number (1–4, each 400 m), running speed (km·h^−1^), cadence (steps·min^−1^), contact time (ms), heart rate (b·min^−1^), age (years), height (cm), body mass (kg), and running experience (years of regular running, self-reported). Contact time was person-mean centred within runner. Heart rate was expressed as a percentage of age-predicted maximum (208 − 0.7 × age) [23] and separated into within-runner and between-runner components. The within-runner component expressed each stride’s heart rate relative to that runner’s own mean, whereas the between-runner component expressed each runner’s mean relative to the group mean. Lap was evaluated as a candidate fixed effect, whereas the random slope for lap allowed the change across the run to differ between runners. Each predictor was then screened individually against a base model with a by-participant random intercept and random slope for lap, which provided the best fit by Akaike Information Criterion (AIC) when compared against models with no random effects and with a random intercept alone. Univariable estimates are reported in Appendix A, Table A1. Predictors with *p* < 0.2 on a chi-squared likelihood ratio test were subsequently entered into a forward and backwards selection process, and only those that improved model fit (*p* < 0.05), confirmed by AIC, were retained.

The variance explained by the by-participant random intercept and random slope for lap was quantified by the difference between marginal *R*^2^_m_ (variance explained by fixed effects only) and conditional *R*^2^_c_ (variance explained by fixed and random effects combined). Model assumptions were evaluated by inspecting residual plots. All analyses were performed in R (version 4.5.0; R Foundation for Statistical Computing, Vienna, Austria), with statistical significance set at *p* < 0.05.

## 3. Results

### 3.1. Participants and Data Screening

Participant characteristics are presented in Table 1. Per-participant scatter plots were inspected during data screening to identify outliers. Participant 21 demonstrated an atypical heart-rate response, remaining below 100 b·min^−1^ throughout the run with no progressive increase. This suggested that the participant completed the run at a substantially lower physiological intensity than intended and therefore did not meet the requirements of the testing protocol. Consequently, this participant was excluded from subsequent analyses. The remaining participants completed the 1600 m run in 7:51 ± 0:48 min:s at a mean speed of 12.5 ± 1.3 km·h^−1^ (Table A2).

### 3.2. Foot Strike Pattern Across 1600 m

**Figure 3:**
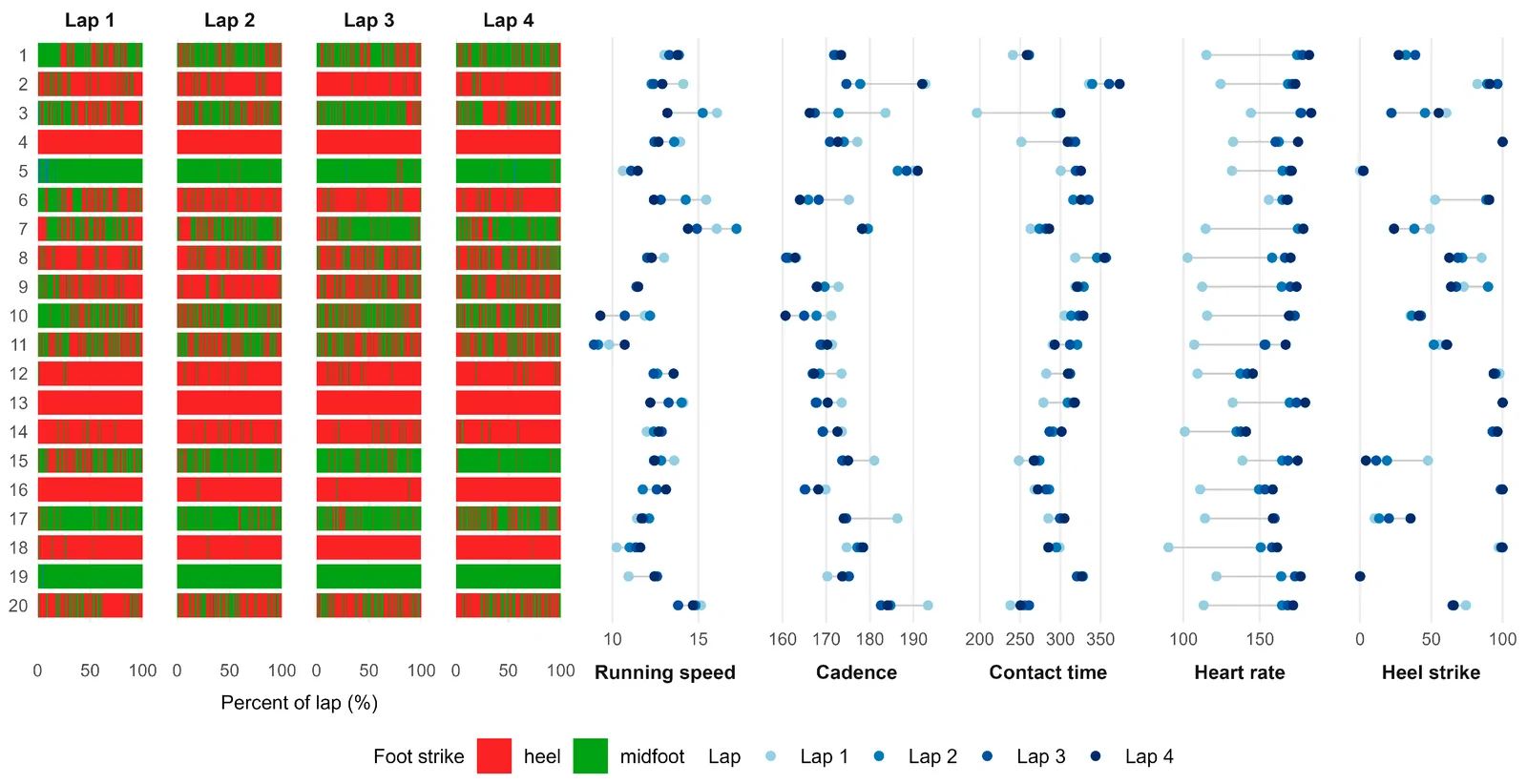
Foot strike pattern, spatiotemporal and physiological measures for 20 runners across four 400 m laps. Strides are coloured by foot strike pattern, and markers show running speed (km·h^−1^), cadence (steps·min^−1^), contact time (ms), heart rate (b·min^−1^) and the percentage of strides classified as heel strikes shaded from light (lap 1) to dark (lap 4).

A total of 12,957 quality strides were retained across the 20 runners, comprising 7828 heel, 5114 midfoot and 15 forefoot strides. Forefoot strides comprised only 0.12% of the total and were contributed by three participants, with 13 of the 15 recorded in a single runner. Foot strike pattern across each lap is shown for each participant in Figure 3. Across runners, the mean percentage of heel strikes was 62.4 ± 33.8%, 61.6 ± 35.8%, 59.8 ± 36.5% and 60.7 ± 35.7% in laps 1 to 4, respectively, with individual values ranging from 0 to 100% in every lap. Patterns varied markedly between participants. Some used a heel-strike pattern almost exclusively throughout the run, whereas others demonstrated a substantial proportion of midfoot strides. Runners also differed in the direction and magnitude of change across laps. The change in heel strike percentage from the first to the fourth lap ranged from a 43.5% decrease to a 38.0% increase, with eight runners increasing, eight decreasing and four unchanged (Figure 3). The change in running speed from the first to the fourth lap ranged from −3.1 to 1.1 km·h^−1^, with 12 runners slowing, seven increasing their speed, and one showing no change (Table A2).

### 3.3. Foot Strike Patterns Across Running and Physiological Characteristics

**Figure 4:**
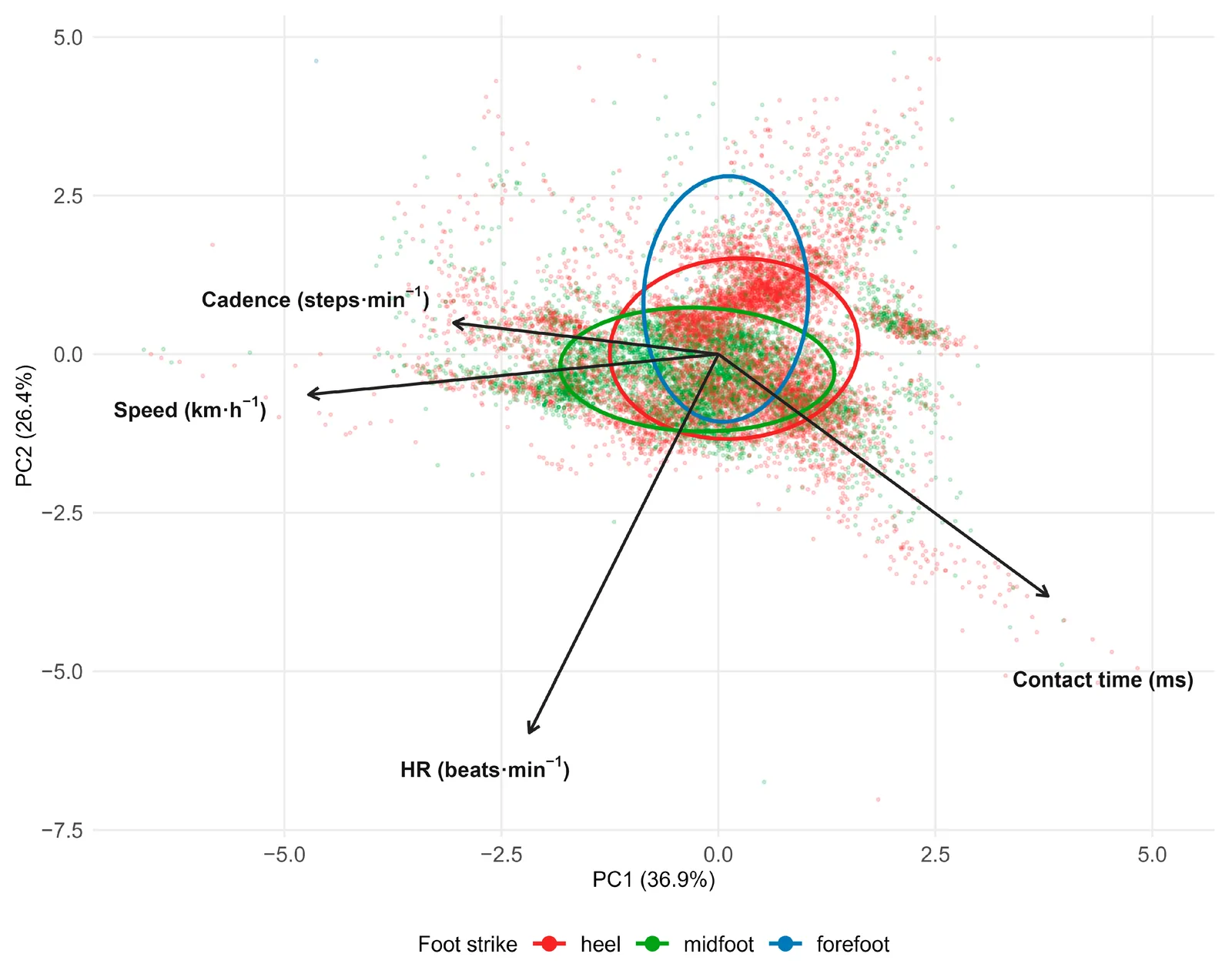
Principal component analysis of speed, heart rate, cadence, and contact time, z-scored, over pooled strides and coloured by foot strike pattern.

The first two principal components explained 36.9% and 26.4% of the total variance, respectively (Figure 4). PC1 was characterised by high loadings for running speed, cadence and contact time and primarily represented gait characteristics, whereas PC2 was characterised by a high loading for heart rate and primarily represented physiological characteristics. Running speed and cadence were positively associated, whereas contact time was inversely related to both variables, indicating that faster running with a higher cadence was associated with shorter ground contact times. Heart rate varied independently of cadence, suggesting that these variables captured different aspects of running performance. Despite these relationships, the heel- and midfoot-strike groups showed substantial overlap, with no principal component clearly separating the strike patterns. Collectively, these variables did not discriminate between heel, midfoot and forefoot strike patterns.

### 3.4. Multivariable Model of Heel Versus Midfoot Striking

Univariable analysis identified five variables for inclusion in the multivariable model (Appendix A, Table A1). Four were retained after selection, whereas cadence was not. The fixed effects explained 18% of the variance in foot strike, whereas the fixed and random effects together explained 81%, indicating that most of the explained variance was attributable to differences between participants (Table 2). Within individuals, for every 1 ms increase in contact time, the odds of heel strike decreased by 1.2% (OR 0.988, 95% CI 0.984–0.991) (Table 2). The odds of heel strike increased by 4.7% for every 1% increase in heart rate relative to a runner’s own mean (OR 1.047, 95% CI 1.034–1.060), and by 27.7% for every 1 km·h^−1^ increase in running speed (OR 1.277, 95% CI 1.177–1.387) (all *p* < 0.001) (Table 2).

## 4. Discussion

The present findings suggest that the proportion of heel strikes does not change systematically during a continuous 1600 m run in recreational female runners. Foot strike was more closely associated with changes in heart rate than with the number of laps completed. Faster running speed and shorter contact time were also associated with heel striking, whereas cadence was not. The first hypothesis was therefore not supported at the group level, although runners differed in the direction and magnitude of change across laps. The second hypothesis was partially supported, as three of the four variables were independently associated with foot strike. Participant-specific effects accounted for most of the variation in foot strike pattern, although within-runner associations remained evident. This suggests that foot strike was influenced more by individual characteristics than by the progression of the run itself.

Running speed, heart rate, and contact time were each associated with foot strike, whereas cadence was not. Two findings differed from previous reports. Faster running in the current study was associated with a greater likelihood of heel striking, in contrast to the shift towards forefoot striking generally reported in studies where speed was imposed [24]. However, that response is not consistent across runners, as many retain their pattern across the same speed range [25]. Likewise, heel striking in the present study was associated with shorter contact times, whereas cross-sectional comparisons often report longer contact times among habitual heel strikers [7]. Both contrasts may reflect how the comparison was made, because the published estimates were drawn between different running populations or across a far wider range of imposed speeds than the runners in the present study, who ran at their own pace.

A further possibility lies in how the runners in the present study ran faster. Running speed can increase through longer steps, higher cadence, or a combination of both. Increasing step length places the foot further ahead of the body’s centre of mass at initial contact, favouring a heel strike while simultaneously reducing ground contact time [26]. Both associations may therefore reflect a single way of running faster rather than two separate effects. This strategy is not universal, however, as world-class 1500 m runners raise speed through cadence rather than step length [9].

The present findings also highlight the importance of distinguishing within-individual from between-individual associations [27]. The heart-rate results illustrate this distinction. Within individuals, increases in heart rate relative to each runner’s own mean were associated with a greater likelihood of heel striking, whereas runners with higher average heart rates were less likely to heel strike. Heart rate rose during the run in nearly every runner, whereas the proportion of heel strikes did not. Contact time offers a possible explanation. Contact time also increased across the run in most runners and was associated with lower odds of heel striking, which may have contributed to the absence of a systematic change in heel-strike proportion. Participant-specific effects accounted for substantially more variation in foot strike than any of the measured biomechanical or physiological variables, and runners also differed in how their foot strike patterns changed throughout the run. Self-reported foot strike matched the measured pattern for fewer than half of the runners. Five runners described themselves as forefoot strikers, whereas forefoot strides comprised a negligible proportion of all strides recorded. This degree of individual variability is consistent with previous reports demonstrating that runners can be identified from their unique running mechanics [28].

This study had several limitations. First, the in-shoe sensors recorded the force between the foot and the shoe rather than the true ground reaction force. Absolute force magnitudes were therefore likely underestimated, although this limitation should not have affected within-runner comparisons [18]. Second, force was sampled at 200 Hz from the right foot only, so between-limb asymmetry could not be examined, and the impact transient was resolved with less temporal detail than a force platform would provide. Any systematic bias arising from the sampling frequency would, however, be expected to be consistent throughout the run, allowing within-runner changes in foot strike to be assessed. Third, forefoot strikes were too few to model, so the findings describe only heel and midfoot patterns. Fourth, maximum heart rate was predicted from age rather than measured, which mainly affects the comparison of heart rate between runners. Finally, the data came from a single session on a synthetic track, where foot strike may differ from that on road and trail surfaces [4]. Each runner also wore her own shoes. Footwear, age and training history were constant within each runner during the run and are therefore unlikely to account for the within-runner associations of contact time and heart rate with foot strike. Although variation in foot strike was predominantly attributable to differences between participants rather than to the progression of the run, a stable individual difference cannot be separated from a day-specific habit, and the runner’s contribution cannot be separated from that of her footwear. Future studies should examine multiple testing sessions using standardised footwear to determine the stability of these individual foot strike patterns.

These findings suggest that assessment across multiple strides or over a sustained period may provide additional information about within-runner variability that is not captured by a single observation. Continuous monitoring may provide a more representative description of running mechanics and better capture the natural variability that occurs during sustained running. The individual differences observed may reflect habitual running technique, footwear or neuromuscular control, which were not measured. Age-related changes in soft-tissue properties, muscle strength and motor control could also contribute, although age was not associated with foot strike in this cohort.

## 5. Conclusions

The findings of this study suggest that foot strike during a continuous 1600 m run varies more between individual runners than across the distance covered. Heel striking became more likely as a runner’s heart rate rose relative to her own average. Faster running and shorter contact time were also associated with heel striking, whereas cadence showed no independent association. Between runners, the heart rate relationship reversed, and differences between runners accounted for most of the observed variation. The proportion of heel strikes did not change systematically across the four laps. These findings suggest that foot strike classified from a single observation may not represent the pattern used across a whole run. Foot strike may therefore be better assessed continuously and for the individual runner, rather than from a single measurement judged against group averages.

## Figures and Tables

**Table 1:** Participant demographics, self-reported foot strike, and mean running characteristics during the continuous 1600 m outdoor run. Summary rows are mean (SD) for all 21 participants and the 20 analysed runners.

Participant	Age (Years)	Height (cm)	Body Mass (kg)	Running Experience (Years)	Contact Time (ms)	Cadence (Steps·min^−1^)	Heart Rate (b·min^−1^)	Heart Rate (% HRmax)	Self-Reported Foot Strike
1	24	152	60.0	2.5	257.0	173.0	175.5	91.8	Midfoot
2	27	170	62.0	4	364.9	181.0	169.6	89.7	Midfoot
3	22	160	66.5	9	292.1	170.1	180.1	93.5	Midfoot
4	24	155	64.5	4	313.3	173.3	165.1	86.4	Midfoot
5	27	155	57.0	10	319.6	191.3	167.1	88.4	Forefoot
6	22	161	62.2	10	327.7	166.6	166.8	86.6	Forefoot
7	31	167	64.6	10	278.9	179.1	175.6	94.2	Heel
8	21	168	65.7	10	349.5	160.9	164.5	85.1	Midfoot
9	22	169	62.8	1.5	325.6	169.0	166.7	86.6	Forefoot
10	26	168	72.0	10	319.0	165.7	168.6	88.8	Midfoot
11	45	160	59.4	2	310.3	169.2	155.4	88.1	Midfoot
12	57	168	63.4	50	305.0	170.2	141.9	84.4	Heel
13	33	167	64.5	20	314.6	168.9	172.4	93.3	Forefoot
14	57	170	56.8	44	297.6	168.7	136.0	80.9	Heel
15	45	169	72.7	30	269.3	174.5	168.5	95.4	Heel
16	23	160	52.8	4	280.3	166.5	151.9	79.2	Midfoot
17	28	166	63.0	23	300.5	175.2	157.0	83.3	Midfoot
18	35	170	68.5	7	291.4	177.2	153.5	83.7	Heel
19	44	167	72.9	2	321.8	175.1	167.4	94.5	Forefoot
20	23	173	64.4	3	255.6	183.9	165.9	86.4	Heel
21 †	47	162	63.5	18	280.4	168.3	74.2	42.4	Midfoot
All participants (*n* = 21)	32.5 (11.8)	164.6 (5.8)	63.8 (5.1)	13.0 (13.6)	303.5 (28.0)	172.7 (7.0)	159.2 (22.3)	85.8 (10.9)	
Analysed (*n* = 20)	31.8 (11.6)	164.8 (5.9)	63.8 (5.2)	12.8 (13.9)	304.7 (28.2)	173.0 (7.1)	163.5 (11.1)	88.0 (4.7)	

† Excluded from analysis.

**Table 2:** Binary logistic mixed-effects multivariable model predicting heel striking (heel = 1, midfoot = 0) across the 1600 m run, with a by-participant random intercept and random slope for lap (12,942 strides, 20 runners).

Factor	Estimate (β)	95% CI (β)	OR	95% CI (OR)	*p*
Intercept (*R*^2^_m_ = 0.179, *R*^2^_c_ = 0.805, ICC = 0.762)	−2.001	−3.761, −0.242			0.026
Contact time (ms, centred)	−0.012	−0.016, −0.009	0.988	0.984, 0.991	<0.001
Heart rate (within-runner, % HRmax)	0.046	0.034, 0.059	1.047	1.034, 1.060	<0.001
Heart rate (between-runner, % HRmax)	−0.395	−0.716, −0.073	0.674	0.489, 0.929	0.016
Speed (km·h^−1^)	0.245	0.163, 0.327	1.277	1.177, 1.387	<0.001

β, log-odds coefficient; CI, confidence interval; OR, odds ratio; HRmax, age-predicted maximum heart rate. A positive β and an OR above 1 indicate greater odds of heel striking. *R*^2^_m_, marginal *R*^2^; *R*^2^_c_, conditional *R*^2^; ICC, intraclass correlation coefficient.

## Data Availability

The data presented in this study are available on reasonable request from the corresponding author.

## Appendix A

**Table A1:** Univariable screening of candidate predictors for heel versus midfoot striking (heel = 1, midfoot = 0). Each predictor was fitted alone against a base model with a by-participant random intercept and a by-participant random slope for lap (12,942 strides, 20 runners).

Factor	Estimate	SE	*z*	Wald *p*	LRT *p*
Lap	0.019	0.109	0.18	0.859	0.861
Speed (km·h^−1^)	0.261	0.041	6.42	<0.001	<0.001
Cadence (steps·min^−1^)	0.008	0.005	1.44	0.151	0.141
Contact time (ms, centred)	−0.012	0.002	−7.54	<0.001	<0.001
Heart rate (within-runner)	0.033	0.006	5.46	<0.001	<0.001
Heart rate (between-runner)	−0.446	0.173	−2.59	0.010	0.034
Age (years)	−0.006	0.074	−0.09	0.931	0.936
Height (cm)	0.053	0.024	2.21	0.027	0.749
Body mass (kg)	−0.225	0.075	−3.01	0.003	0.219
Running experience (years)	0.036	0.077	0.47	0.637	0.642

SE, standard error; LRT, likelihood ratio test. Likelihood ratio tests compare each predictor against the base model on 1 degree of freedom. Predictors with *p* < 0.2 were entered into the selection process.

**Table A2:** Running performance for each participant during the continuous 1600 m outdoor run, completed as four 400 m laps (*n* = 20). The bottom row is mean (SD).

Participant	Lap 1 (km·h^−1^)	Lap 2 (km·h^−1^)	Lap 3 (km·h^−1^)	Lap 4 (km·h^−1^)	1600 m Time (min:s)	Mean Speed (km·h^−1^)
1	13.7	13.0	13.2	13.2	7:14	13.3
2	13.2	12.1	12.0	12.0	7:48	12.3
3	15.8	13.5	13.0	13.3	6:57	13.8
4	13.5	12.5	13.0	13.8	7:17	13.2
5	10.9	11.0	11.1	12.0	8:33	11.2
6	15.5	13.5	12.0	12.4	7:16	13.2
7	15.5	15.3	14.0	14.0	6:33	14.6
8	12.5	12.0	12.0	12.0	7:55	12.1
9	11.6	11.3	11.2	11.2	8:28	11.3
10	12.5	11.1	10.4	10.3	8:43	11.0
11	9.7	8.8	9.9	10.3	9:59	9.6
12	13.2	12.2	12.3	12.6	7:38	12.6
13	13.0	13.7	13.1	13.1	7:16	13.2
14	11.7	11.5	11.6	11.7	8:15	11.7
15	13.6	12.1	12.2	12.0	7:43	12.5
16	11.7	11.9	12.3	12.6	7:55	12.1
17	11.8	11.9	11.4	11.7	8:12	11.7
18	10.7	11.1	11.4	11.4	8:36	11.2
19	12.3	12.4	12.4	12.4	7:45	12.4
20	15.0	13.5	13.6	14.5	6:48	14.1
Mean (SD)	12.9 (1.7)	12.2 (1.4)	12.1 (1.0)	12.3 (1.1)	7:51 (0:48)	12.5 (1.3)
